# Pilot Study of Urinary Biomarkers of Phytoestrogens, Phthalates, and Phenols in Girls

**DOI:** 10.1289/ehp.9488

**Published:** 2006-10-19

**Authors:** Mary S. Wolff, Susan L. Teitelbaum, Gayle Windham, Susan M. Pinney, Julie A. Britton, Carol Chelimo, James Godbold, Frank Biro, Lawrence H. Kushi, Christine M. Pfeiffer, Antonia M. Calafat

**Affiliations:** 1 Mount Sinai School of Medicine, Division of Environmental Health Sciences, Department of Community and Preventive Medicine, New York, New York, USA; 2 Division of Environmental and Occupational Disease Control, California Department of Health Services, Oakland, California, USA; 3 Department of Environmental Health, University of Cincinnati College of Medicine, Cincinnati, Ohio, USA; 4 Division of Adolescent Medicine, Cincinnati Children’s Hospital Medical Center, Ohio, USA; 5 Division of Research, Kaiser Permanente, Oakland, California, USA; 6 Division of Laboratory Sciences, National Center for Environmental Health, Centers for Disease Control and Prevention, Atlanta, Georgia, USA

**Keywords:** biomarkers, children, exposure, phenols, phthalates, phytoestrogen, urine

## Abstract

**Background:**

Hormonally active environmental agents have been measured among U.S. children using exposure biomarkers in urine. However, little is known about their variation by race, age, sex, and geography, and no data exist for newly developed biomarkers.

**Objective:**

Our goal was to characterize relevant, prevalent exposures for a study of female pubertal development.

**Methods:**

In a pilot study among 90 girls from New York City, New York, Cincinnati, Ohio, and northern California, we measured 25 urinary analytes representing 22 separate agents from three chemical families: phytoestrogens, phthalates, and phenols. Exposures occur chiefly from the diet and from household or personal care products.

**Results:**

Participants represented four racial/ethnic groups (Asian, black, Hispanic, white), with mean age of 7.77 years. Most analytes were detectable in > 94% of samples. The highest median concentrations for individual analytes in each family were for enterolactone (298 μg/L), monoethylphthalate (MEP; 83.2 μg/L), and benzophenone-3 (BP3; 14.7 μg/L). Few or no data have been reported previously for four metabolites: mono(2-ethyl-5-carboxypentyl) phthalate, triclosan, bisphenol A (BPA), and BP3; these were detected in 67–100% of samples with medians of 1.8–53.2 μg/L. After multivariate adjustment, two analytes, enterolactone and BPA, were higher among girls with body mass index < 85th reference percentile than those at or above the 85th percentile. Three phthalate metabolites differed by race/ethnicity [MEP, mono(2-ethylhexyl) phthalate, and mono-3-carboxypropylphthalate].

**Conclusions:**

A wide spectrum of hormonally active exposure biomarkers were detectable and variable among young girls, with high maximal concentrations (> 1,000 μg/L) found for several analytes. They varied by characteristics that may be relevant to development.

Effects of hormonally active environmental agents on early child development have been of concern, as knowledge has become available about their biological activity and about widespread exposure. For agents that are short-lived in the body (i.e., rapidly metabolized and/or eliminated), assessment of exposure biomarkers in urine is usually preferred for several reasons: The metabolites are readily detectable in urine, urine is easy to collect, and urine generally has higher concentrations of polar metabolites than other biologic media. Although exposures to many of these agents have been characterized in children [Centers for Disease Control and Prevention (CDC) 2005], little is known about variation of these exposure biomarkers by race, age, body mass index (BMI), and sex.

The Breast Cancer and the Environment Research Centers (BCERC) are a consortium established by the National Institute of Environmental Health Sciences and the National Cancer Institute to elucidate influences of environmental factors on early pubertal development in girls, and thereby possible future risk for breast cancer and other chronic diseases among women. For this purpose, the study design employs biomarkers to assess a variety of environmental exposures. The highest-priority urinary exposure biomarkers identified by the BCERC consortium are phytoestrogens, phthalate acids, and phenols. Agents in these groups were selected because they possess hormonal activity that may be agonistic or antagonistic (Fenton 2006; Rajapakse et al. 2002; Sohoni and Sumpter 1998); they have been detected at sufficiently high concentrations to constitute a potential risk (CDC 2005); and they were known or expected to have adequate interindividual variability to serve as exposure markers. Exposures to these chemicals occur chiefly through the diet and use of household or personal care products (Table 1) (Calafat et al. 2005; CDC 2005; Duty et al. 2005). The CDC has previously reported concentrations in child participants in the National Health and Nutrition Examination Survey (NHANES) for some biomarkers (CDC 2005). However, no data are available in children for certain phenols, including bisphenol A (BPA), a chemical with hormonal activity relevant to pubertal development (vom Saal and Hughes 2005). In addition, prevalence and variability of these exposure biomarkers have not been described among young girls, and it is not known how these exposures may vary by race, geographic location, or age. In this report we provide initial information on levels of biomarkers for three chemical families of primary interest—phytoestrogens, phthalates, and phenols—and on their distribution by demographic factors among a subsample of the BCERC study population.

## Methods

Participants in this pilot study were among the first children enrolled at three BCERC centers. We are recruiting approximately 1,200 girls 6–8 years of age into a longitudinal study, with the aim of following pubertal development from its earliest stages through menarche. Eligibility includes age 6–8 years, female sex, and no underlying endocrine medical conditions. All sites obtained informed consent from parent or guardian and child assent, approved by each institution’s institutional review board. Study designs and methods were standardized for most but not all components, because each center retained some unique scientific aims, and their recruitment began at different dates. Mount Sinai School of Medicine (MSSM) is recruiting black and Latina girls from clinics, schools, and community centers in East Harlem in New York City; the sample population from the University of Cincinnati/Cincinnati Children’s Hospital (Cincinnati) is recruited from and examined at schools or through a breast cancer registry; the Kaiser Permanente Northern California (Kaiser) study group is recruited from the Kaiser health maintenance organization (HMO) membership in the San Francisco Bay area. At all sites, a baseline questionnaire was completed by the girl’s parent or guardian (usually the mother) that included a detailed medical history, product use and exposures, exercise, diet, and demographic variables. Self-reported race/ethnicity included white, black, American Indian, Pacific Islander, or Asian, as well as Hispanic ethnicity. There were 10 Hispanics of Mexican origin at MSSM who did not report a race but who were identified by interviewers as Native Americans by ascertaining child and parental birthplace and native language (Mexican Indians). For the purposes of this report, race/ethnicity was classified as black (including black Hispanic), non-black Hispanic, non-Hispanic white, or non-Hispanic Asian.

Height and weight were measured using calibrated scales and stadiometers by interviewers who had been trained and certified uniformly across all three sites. BMI was calculated as weight/height-squared (kilograms per square meter) and then classified as < 85th national percentile, age- and sex-specific, or ≥ 85th percentile (Himes and Dietz 1994), using CDC growth charts (CDC 2000). In this CDC data set, the 85th percentile BMI cut points for girls in the first month of 6, 7, and 8 years of age are 17.100, 17.626, and 18.317 kg/m^2^, respectively. Urine specimens were collected at the time of the baseline examination or in a 6-month follow-up visit (Cincinnati). MSSM and Kaiser collected spot specimens at baseline, and Cincinnati collected early morning voids. Each center submitted 30 urine samples to CDC for determining the concentrations of phthalate metabolites, phenols, phytoestrogens, and creatinine (to normalize for urine dilution). Samples from MSSM and Cincinnati were selected randomly from the samples donated before December 2005 with at least 40 mL of urine. Kaiser sent the first 30 samples collected with sufficient volume. The study size was limited by budgetary constraints and by the need to conduct the pilot study at an early stage of recruitment.

Laboratory techniques used by CDC for measuring the selected exposure biomarkers in urine have been published. Briefly, metabolites are deconjugated enzymatically, because these agents are excreted almost entirely as conjugated metabolites. Matrix removal and analyte enrichment are accomplished by solid phase extraction, and instrumental analysis is done with high performance liquid chromatography–tandem mass spectrometry using isotope dilution quantification (Kato et al. 2005; Kuklenyik et al. 2004; Rybak et al. 2006; Ye et al. 2005a). The laboratory is certified according to the Clinical Laboratories Improvement Act, and procedures incorporate quality control (QC) measures to ensure accuracy and precision of results, including annual proficiency testing compliance (Norrgran et al. 2006). A laboratory batch must meet quality control criteria, including acceptable blanks, or the batch is entirely reanalyzed. Results are blank-corrected. Enterodiol data for two girls were not available because these results did not fulfill the quality assurance/quality control requirements. Creatinine was measured using an enzymatic reaction on a Roche Hitachi 912 chemistry analyzer (Roche Hitachi, Basel Switzerland).

We performed statistical analyses using SAS (version 9.1.3 for PC; SAS Institute Inc., Cary, NC). Because of the unequal distribution of characteristics among sites, we first used nonparametric methods to examine variation of exposure biomarker concentrations in relation to study characteristics. For analytes detected in > 60% of samples, we then performed multivariate analyses adjusting for age, race/ethnicity, site, BMI, and season of sample collection using the general linear model (GLM) procedure, which accommodates unbalanced designs. For age, we computed Spearman correlations with each biomarker. Using the Kruskal-Wallis test of rank sums (NPAR1WAY procedure with Wilcoxon option), we compared medians of the creatinine-corrected concentrations (micrograms per gram creatinine) by race/ethnicity, site, BMI, and season (coded as Summer = June, July, August vs. other seasons; use of several products listed in Table 1 was considered likely to vary by season). For exposure biomarkers whose medians exhibited differences with respect to these characteristics [see Supplemental Materials, Table 1 (http://www.ehponline.org/docs/2006/9488/suppl.pdf)], we then examined whether the multivariate geometric means were significantly different across characteristic levels using the LSMEANS option of the GLM procedure. Four Asians were retained in the multivariate analyses, with racial/ethnic differences reported only for blacks, Hispanics, and whites but not Asians. In parametric analyses, we used log-transformed values of the urinary metabolite concentrations to normalize the distribution and we substituted the value LOD/√2 for results below the limit of detection (LOD) following the CDC practice (Wolff et al. 2005).

## Results

Participants represented four racial/ethnic groups (Asian, black, Hispanic, white) and three geographic locations (New York City, Cincinnati metropolitan area, and the San Francisco Bay area of California; Table 2). Mean age was 7.77 years at date of sample collection (range, 6.4–9.2). Samples collected at Kaiser were almost all from 7-year-old girls (29 of 30); there were two 6-year-old girls from Cincinnati and nine from MSSM. Race/ethnicity varied by site, with Kaiser and Cincinnati girls mainly white (> 60%) or black, and with no whites at MSSM. All four Asians were from Kaiser, and most Hispanics (18 of 22) were from MSSM. Compared with national data (CDC 2000), 32% of girls were ≥ 85th percentile of BMI, and the distributions of BMI within sites were similar. Samples from Kaiser were all collected in summer; no samples from Cincinnati and nine from MSSM were collected in summer.

Eighteen of the 25 analytes were detected in at least 94% of the samples (Table 3). Phytoestrogens as a group had the highest concentrations (e.g., median 298 μg/L for enterolactone), and all six phytoestrogens were detected in > 98% of samples. Phthalate metabolites were intermediate in concentration, with 9 of the 10 biomarkers detected in > 94% of samples. Phenols had the lowest concentrations and were least detected (only 3 of the 9 were detected in > 94% of samples). Seventeen exposure biomarkers had medians > 10 μg/L (10 ppb), and six had medians > 50 μg/L. Four phytoestrogens, four phthalates, and two phenols had maximum values > 1,000 μg/L (1 ppm). The ranges for 10 of 25 exposure biomarkers encompassed at least 3 orders of magnitude (e.g., 1–1,000 μg/L). The highest individual biomarker measurement was for benzophenone-3 (BP3; 26,700 μg/L). To verify that the very high BP3 urinary measurements reflected absorbed dose rather than surface contamination during sample collection, storage, or analysis, we measured free (i.e., unbound) BP3 in the nine samples having concentrations >1,000 μg/L. The concentrations of unbound BP3 were minimal, < 1–20 μg/L (data not shown), consistent with excretion of absorbed BP3 as conjugated species (Ye et al. 2005b).

Multivariate adjusted concentrations of creatinine-corrected exposure biomarkers (geometric means) are presented in Table 4 according to race/ethnicity, geographic site, BMI, and season of collection; included are the 20 analytes that were detected in at least 60% of samples. Differences in the medians (unadjusted) by characteristics are shown in the Supplemental Material, Table 1 (http://www.ehponline.org/docs/2006/9488/suppl.pdf). Compared with the unadjusted values, there were fewer significant associations in the multivariate models for race (5 vs. 8), site (3 vs. 9), and season (1 vs. 6). Enterolactone and BPA differed significantly with regard to BMI (< 85th percentile vs. ≥ 85th percentile). The adjusted geometric means of three phthalate metabolites varied by race/ethnicity, with whites having lower concentrations of mono(2-ethylhexyl) phthalate (MEHP) and monoethyl phthalate (MEP) but higher mono(3-carboxypropyl) phthalate (MCPP). Among the phenols, 2,5-dichlorophenol (25DCP) was higher in blacks than whites, and BP3 was higher in whites. *O*-Desmethylangolensin (*O*-DMA), 25DCP, and 2,4-dichlorophenol (24DCP) differed across the three study sites. BP3 was higher in samples collected in summer. Patterns by race and season for MEHP, MEP, MCPP, 25DCP, and 24DCP remained the same if the geometric means were adjusted for age, race, BMI, and season but not for site. When the correlation between age as a continuous variable and each analyte was examined, it was significantly associated only with equol (micrograms equol/grams creatinine; *r_S_* −0.26, *p* = 0.013). In the multivariate model for equol [ln, micrograms per gram creatinine], the beta for age (years) was −0.44 (*p* = 0.029, adjusted for race/ethnicity, geographic site, BMI, and season of collection).

The strongest correlations between individual biomarkers within a family were seen among those arising from the same parent compound (e.g., *r_S_* = 0.79–0.99 among four di(2-ethylhexyl) phthalate [DEHP] metabolites: mono(2-ethyl-5-carboxypentyl) phthalate [MECPP], mono(2-ethyl-5-hydroxyhexyl) phthalate [MEHHP], (2-ethyl-5-oxohexyl) phthalate [MEOHP], and MEHP; data not shown). We computed correlations between creatinine and the urinary exposure biomarkers to examine the appropriateness of creatinine-corrections for dilution. The lowest correlations were seen for BP3 (*r_S_* = −0.03, *p* = 0.758) and *O*-DMA (*r_S_* = 0.24, *p* = 0.022); correlations of creatinine with other biomarkers were fairly strong (*r_S_* > 0.3, *p* < 0.01; data not shown). Associations of phthalate metabolites with creatinine were stronger (*r_S_* = 0.50–0.72) than for phytoestrogens (*r_S_* = 0.33–0.52, not including *O*-DMA) and phenols (*r_S_* = 0.42–0.54 for triclosan, 25DCP, BPA, and 24DCP). Because BP3 was not related to urinary creatinine, it may be inappropriate to correct for dilution using creatinine (Hauser et al. 2004; Miller et al. 2004). When we examined BP3 in relation to the characteristics in Table 4 using the concentration as micrograms per liter (uncorrected for creatinine), we obtained almost identical results. The range of creatinine was 7.6–255 mg/dL with 9 samples < 20 mg/dL, which could potentially influence the data in Tables 3 and 4 by overinflating the creatinine-corrected values. We examined the distribution of values for the nine low-creatinine samples in those exposure biomarkers that varied significantly by the factors in Table 4; they were fairly evenly distributed in terms of concentrations, and excluding them from the multivariate adjusted models did not alter the differences seen in the exposure biomarkers with regard to characteristics described above. In addition, in models where biomarkers were not creatinine-corrected (micrograms per liter), we observed results similar to those in Table 4, except that two associations were not significant (BPA with BMI, *p* = 0.11; and 24DCP by site, *p* = 0.13).

## Discussion

This pilot study of peripubertal girls examined urinary biomarkers of exposures among three chemical families that possess known or likely hormonal activity. Biomarkers from these families appear to be ubiquitous, have wide variability, and show relatively high urinary concentrations in 6- to 9-year-old girls, suggesting that they are suitable for study of exposure–outcome associations related to puberty. Among the 25 exposure biomarkers measured in this study, we had initially identified eight compounds as high priority for the epidemiologic study, based on criteria of having prevalent, high exposure levels, toxicologic relevance, and exposure biomarker reliability. These included three phytoestrogens (enterolactone, daidzein, genistein), three phthalate metabolites (mono-*n*-butyl phthalate [MBP], monobenzyl phthalate [MBzP], MEP), and two phenols (BPA, nonylphenol). Seven of these biomarkers were detected in at least 94% of samples, and the ranges of concentrations were wide, from the LOD (< 1) to > 26,000 μg/L (minimum–maximum). Nonylphenol was not determined because CDC as yet has no optimal biomarker for this compound (Calafat et al. 2005).

Levels of phytoestrogen and phthalate metabolites in this study were similar to those reported in the NHANES 2001–2002 children (CDC 2005), although enterodiol appeared to be higher and MBzP and monomethyl phthalate (MMP) lower in our study population. MECPP and MEP had the highest levels of the 10 phthalate metabolites measured. MECPP, a DEHP metabolite, has not been previously reported in NHANES nor in school-age children. The relationship of equol with age could be of interest, but it was the only analyte related to age. This association could also be attributable to population characteristics that can be explored in the future, such as diet. Two biomarkers (enterolactone and BPA) varied by BMI, and three phthalates differed by race. Relationships of phthalates with race/ethnicity were quite similar to those reported for all ages in the NHANES 2001–2002—for example, MEP and MEHP were highest in blacks and MCPP highest in whites (CDC 2005) (the CDC report does not provide race-specific data for children). One biomarker varied by season (BP3) and three by site (*O*-DMA, 25DCP, 24DCP), differences that may reflect diverse exposures; alternatively, these observations may be attributed to the unequal distribution of characteristics by site, a notion supported by the finding that both BP3 and 25DCP also differed by race. Differences by race and BMI may also be attributed to other confounding factors, including socioeconomic status (SES), that were not available for consideration. SES may affect body size, dietary habits, and product use, for example.

Among the four DEHP metabolites measured, MEHP differed significantly by race after multivariate adjustment, whereas the trends for the three oxidative metabolites of DEHP were similar but not statistically significant (Table 4). It is possible that we did not detect significant racial/ethnic trends for the three DEHP oxidative metabolites because of limited sample size. It is also possible for MEHP to arise from sample contamination or hydrolysis, whereas the oxidative metabolites must be endogenous; however, this is unlikely to explain racial/ethnic variability. Alternatively, although overall exposures to DEHP may have been fairly uniform, recent ambient exposures to DEHP may have varied by race/ethnicity in this population. In another study, racial/ethnic differences in MEP levels were attributed to greater use of cologne by blacks and Hispanics (Duty et al. 2005). If recent exposures were responsible for the racial/ethnic differences in our data, a possibility would be that MEHP as the first metabolite formed might differ by race/ethnicity, reflecting mainly the most recent exposures. Another possible explanation is that there may be racial/ethnic variability in the primary but not the secondary DEHP metabolic routes. MEHP is the initial metabolite of DEHP, and it can undergo further oxidation through separate pathways, producing MECPP by one route and MEHHP and MEOHP by another. Racial/ethnic differences in the secondary pathways could be smaller than for MEHP formation. In addition, the oxidative metabolites have longer half-lives [10–15 hr vs. 5 hr for MEHP (Koch et al. 2006)], possibly constituting a more integrated measure of long-term exposures that vary less by race/ethnicity.

The high correlations observed among four DEHP metabolites signify a common source of exposure from the parent compound (Silva et al. 2006). The proportion of MECPP (> 50% of the total of four metabolites reported here) is consistent with previous research suggesting that infants have a much higher proportion of MECPP (66%) than adults (32%) (Silva et al. 2006). Among other phthalate metabolites, MCPP and MEP, which differed significantly by race but in different directions, were not highly correlated with each other (*r_S_* = 0.33), supporting different product origins and environmental exposures for these agents.

The relationships of the urinary exposure biomarkers to creatinine levels are of interest. One site collected early-morning (but not first-morning) voids, which may reflect different accumulated exposures than daytime spot samples that creatinine correction cannot resolve entirely. However, collection time is not likely to bias our exposure measures, because the adjusted means differed by site for only three analytes in two chemical families, and specific exposures are as likely to explain these findings as collection time. As recognized by others, care must be taken in normalizing urinary analytes for dilution because the excretory mechanisms are not the same for creatinine and certain chemicals (Hauser et al. 2004; Miller et al. 2004). This possibility is evident in our study where the correlations between creatinine and endocrine disruptor biomarkers varied, from zero (BP3) to 0.72 (mono-isobutyl phthalate [MiBP]), suggesting that excretion or metabolic capacity may affect these associations, possibly related to the amount of bound (e.g., glucuronidated) versus unbound metabolites. However, in agreement with previous research (Ye et al. 2005b), our data show that even at very high concentrations, BP3 is excreted mostly conjugated in urine; in contrast, MEP is mostly unconjugated (Silva et al. 2003). Unlike creatinine, glucuronide conjugates are actively excreted by tubular secretion, which may explain in part the low correlation of BP3 with creatinine as well as the wide range of correlations between creatinine and these biomarkers (see Hauser et al. 2004 and references therein). An alternative to correcting for urinary concentration is urinary specific gravity (Hauser et al. 2004; Miller et al. 2004), but this measurement was not performed on our samples.

The differences in exposure biomarkers by race/ethnicity and BMI are potentially relevant to pubertal development which is known to be associated with these characteristics (Herman-Giddens et al. 1997). Furthermore, the associations of enterolactone and BPA with BMI and of phthalate metabolites with race are notable because these biomarkers did not vary by other factors in our data. Although the differences we observed are suggestive only, because the sample size is small and unbalanced with regard to some characteristics, we used a conservative approach, considering only a limited number of *a priori* comparisons. Furthermore, the findings for phthalate metabolites were consistent with earlier reports. In general, concentrations of the 25 urinary exposure biomarkers we measured are far higher than those of more widely studied environmental agents such as 1,1′-dichloro-2,2′-bis(4-chlorophenyl)ethylene (DDE) and elemental lead (CDC 2005). In addition, some of these agents have relatively potent hormonal activity. In yeast assays, for example, BPA and butylbenzyl phthalate (the parent compound of MBzP) have greater antiandrogenic and estrogenic activity than DDE (Sohoni and Sumpter 1998). However, the proportional biological effects of these exposures in humans are not known. Several exposure biomarkers reported here have not previously been measured in children nor in different parts of the United States. On the basis of this pilot study, we are considering the potential relevance to child development of additional exposures that were not originally planned for study, and we are exploring alternatives to creatinine correction. If we identify any of these biomarkers as either protective or detrimental in terms of child maturation, the levels of these chemicals in the body may be modifiable because they are derived from the diet or ambient environment or from personal product use. Additional studies are under way to identify sources of these agents in our population and to assess the temporal variation of urinary metabolites among children.

## Figures and Tables

**Table 1:** Biomarkers, their parent compounds, and examples of their environmental sources.

Chemical class and examples	Abbreviation	Main parent compound (if applicable)	Exposure sources
Phytoestrogens			Dietary intake
Isoflavones (genistein, daidzein)			Soy products, including processed meats, meat substitutes, breads, and protein food bars
Lignans (enterolactone)			Flax, seeds, grains
Phthalate metabolites			Industrial and personal product additives
Mono(2-ethylhexyl) phthalate	MEHP	Di(2-ethylhexyl) phthalate (DEHP)	Soft plastic including tubing, especially polyvinyl chloride (PVC; e.g., sometimes present in clear food wrap)
Mono(3-carboxypropyl) phthalate	MCPP	Di-*n*-octyl phthalate	Soft plastic
Monobenzyl phthalate	MBzP	Butylbenzyl phthalate	Vinyl flooring, adhesives
Monoethyl phthalate	MEP	Diethyl phthalate (DEP)	Shampoo, scents, soap, lotion, cosmetics
Monomethyl phthalate	MMP	Dimethyl phthalate (DMP)	Insect repellant, plastic
Mono-*n*-butyl phthalate, mono-isobutyl phthalate	MBP, MiBP	Dibutyl phthalate (DBP), diisobutyl phthalate (DiBP)	Adhesives, caulk, cosmetics
Phenols			Commercial and personal products and additives
Bisphenol A	BPA		Polycarbonate containers and coatings (cans, cups), dental sealant
Benzophenone-3(2-hydroxy-4-methoxy-benzophenone)	BP3		Sunscreen
Triclosan [5-chloro-2-(2,4-dichlorophenoxy)phenol]	TRCS		Microbicide in cleaning fluids
2,4-Dichlorophenol and trichlorophenols	24DCP, 245TCP, 246TCP	Phenoxy- and other derivatives	Herbicides
2,5-Dichlorophenol	25DCP	4-dichlorobenzene	Mothballs
*ortho*-Phenylphenol	*o*-PP		Fungicide
4-*tert*-Octylphenol	4-*t*-OP		Detergent surfactant

**Table 2:** Characteristics of the study population, BCERC pilot study, 2004–2005.

		No. per site		
Characteristic	No. (%)	MSSM	Cincinnati	Kaiser
Age at sample collection (years)^a^				
6.0–6.9	11 (12.2)	9	2	0
7.0–7.9	57 (63.3)	9	19	29
≥ 8.0	22 (24.4)	12	9	1
Race/ethnicity				
Asian	4 (4.4)	0	0	4
Black	26 (28.9)	12	9	5
Hispanic	22 (24.4)	18	1	3
White	38 (42.2)	0	20	18
Site				
Cincinnati	30 (33.3)			
MSSM	30 (33.3)			
Kaiser	30 (33.3)			
BMI for age^b^				
< 85th percentile	61 (67.8)	21	19	21
≥ 85th percentile	29 (32.2)	9	11	9
Collection time^c^				
June–August	39 (43.3)	9	0	30
Other months	51 (56.7)	21	30	0

a Some samples were collected at a visit 6 months after the baseline visit.

b Available: http://www.cdc.gov/growthcharts/ (CDC 2000).

c October 2004 to September 2005.

**Table 3:** Distribution of BCERC phytoestrogen, phthalate, and phenol and biomarkers for all sites combined, 2004–2005.

					Range (μg/L)					
Analyte	No.	No. > LOD	LOD (μg/L)	Percent > LOD	Low	High	Median (μg/L)	Geometric mean [GSD (μg/L)]	Geometric mean [GSD (μg/gC)]	NHANES 50th percentile^a^ (μg/L)
Phytoestrogens										
Enterolactone	90	90	0.3	100.0	4.6	6730.0	298.0	269.0 (4.1)	420.0 (3.7)	329.0
Daidzein	90	90	0.3	100.0	2.4	9690.0	98.0	112.0 (5.7)	175.0 (5.2)	72.7
Enterodiol	88	88	0.3	100.0	1.0	548.0	63.7	54.8 (3.3)	86.5 (2.8)	35.4
Genistein	90	90	0.3	100.0	1.2	5360.0	50.1	60.4 (5.4)	94.3 (4.7)	31.5
Equol	90	89	0.3	98.9	0.2	485.0	10.5	10.9 (4.1)	17.0 (3.5)	13.6
*O*-DMA	90	89	0.2	98.9	0.1	3210.0	5.7	5.7 (9.3)	8.9 (8.6)	5.7
Phthalates										
MECPP^b^	90	90	0.25	100.0	5.9	2260.0	53.2	50.3 (3.1)	78.5 (2.5)	
MEHHP^b^	90	90	0.32	100.0	1.4	1699.0	25.9	28.0 (3.4)	43.8 (2.7)	32.9
MEOHP^b^	90	90	0.45	100.0	1.3	1070.0	17.8	18.8 (3.3)	29.3 (2.6)	22.6
MEHP^b^	90	85	0.90	94.4	0.6	110.0	3.2	3.3 (3.0)	5.2 (2.7)	4.4
MEP	90	90	0.40	100.0	5.3	2580.0	83.2	75.7 (3.9)	118.0 (3.1)	71.9
MBP	90	88	0.40	97.8	0.3	363.0	37.4	28.2 (3.4)	44.1 (2.4)	32.4
MBZP	90	89	0.11	98.9	0.1	191.0	22.2	18.4 (4.0)	28.7 (2.8)	37.0
MIBP	90	87	0.26	96.7	0.2	144.0	7.7	7.1 (3.6)	11.1 (2.5)	4.4
MCPP	90	90	0.16	100.0	0.4	76.9	6.3	6.1 (2.9)	9.5 (2.1)	6.6
MMP	90	18	1.00	20.0	< LOD	15.6	< LOD	< LOD	< LOD	1.8
Phenols										
BP3	90	86	0.34	95.6	< 0.2	26700.0	14.7	19.7 (14.6)	30.8 (15.5)	82.3
Triclosan	90	61	2.27	67.8	< 1.6	956.0	7.2	10.9 (6.5)	17.1 (5.5)	12.5
25DCP	90	88	0.12	97.8	< 0.1	3120.0	7.1	9.0 (11.9)	14.0 (9.9)	3.1
BPA	90	85	0.36	94.4	< 0.3	54.3	1.8	2.0 (3.2)	3.0 (3.0)	2.4
24DCP	90	70	0.17	77.8	< 0.1	92.7	0.9	0.9 (5.3)	1.4 (4.3)	0.6
246TCP	90	22	0.50	24.4	< LOD	6.1	< LOD	< LOD	< LOD	0.3
245TCP	90	21	0.10	23.3	< LOD	1.2	< LOD	< LOD	< LOD	0.1
4*-t*-OP	90	5	0.17	5.6	< LOD	0.4	< LOD	< LOD	< LOD	0.5
*o*-PP	90	3	0.10	3.3	< LOD	2.5	< LOD	< LOD	< LOD	0.4
Creatinine (mg/dL)	90				7.6	254.8	76.2			

Abbreviations: GSD, geometric standard deviation; μg/gC, the urinary concentration (μg/L) corrected for creatinine (g/L).

a NHANES 50th percentile values for phthalates and phytoestrogens for 6- to 11-year-old children obtained from the Third National Report on Human Exposure to Environmental Chemicals (CDC 2005); 50th-percentile values for phenols obtained from Ye et al. (2005a).

b Derived from DEHP.

**Table 4:** Geometric means (μg/g creatinine) of phytoestrogen, phthalate, and phenol biomarkers (those ≥ 60% LOD) adjusted for age, race, site, BMI, and season, 2004–2005.

	Race/ethnicity				Site			BMI for age		Season of sample collection	
Total (*n* = 90)	Asian (*n* = 4)	Black (*n* = 26)	Hispanic (*n* = 22)	White (*n* = 38)	Cincinnati (*n* = 30)	MSSM (*n* = 30)	Kaiser (*n* = 30)	< 85th reference percentile (*n* = 61)	≥ 85th reference percentile (*n* = 29)	Summer (*n* = 39)	Other season (*n* = 51)
Phytoestrogens											
Enterolactone	174.0	414.0	388.0	287.0	292.0	185.0	495.0	513.0	174.0*	236.0	378.0
Daidzein	53.4	166.0	188.0	205.0	154.0	69.8	234.0	115.0	161.0	97.1	190.0
Enterodiol	157.0	58.5	81.5	97.1	113.0	65.4	107.0	107.0	79.9	99.9	85.4
Genistein	52.2	83.7	96.8	111.0	81.8	56.3	123.0	68.4	100.0	55.5	123.0
Equol	12.1	10.7	20.2	17.9	17.9	20.1	8.9	16.7	12.9	10.7	20.2
*O*-DMA	0.9	12.0	6.6	7.5	7.1	1.6	10.1*	6.7	3.5	3.5	6.8
Phthalates											
MECPP^a^	119.0	80.7	98.1	69.2	91.5	111.0	71.5	86.6	93.4	100.0	80.9
MEHHP^a^	58.2	55.0	43.3	42.0	48.3	64.8	37.8	43.0	56.1	53.0	45.5
MEOHP^a^	37.3	33.8	29.0	29.0	31.6	41.5	25.2	28.8	35.8	33.6	30.6
MEHP^a^	8.3	7.9	4.5	4.3*	5.9	7.2	5.0	5.5	6.5	6.4	5.6
MEP	66.1	194.0	231.0	73.5*	161.0	100.0	111.0	102.0	144.0	140.0	105.0
MBP	48.2	39.0	50.9	44.4	46.7	49.7	40.3	43.3	47.6	42.1	48.9
MBZP	8.0	24.2	33.2	35.7	24.9	22.3	18.9	20.4	23.5	21.1	22.7
MiBP	15.3	10.1	11.3	11.8	10.4	14.6	11.3	10.8	13.3	10.2	14.1
MCPP	5.5	6.5	9.6	12.0*	9.3	7.8	7.2	8.8	7.3	8.5	7.6
Phenols											
BP3	42.2	21.7	14.9	92.7*	14.5	25.6	101.0	26.9	41.8	83.8	13.4*
Triclosan	6.7	22.0	16.3	14.3	21.2	14.8	8.0	15.8	11.7	13.3	13.9
25DCP	16.2	28.5	15.6	8.8*	6.7	85.0	7.1*	12.3	20.6	10.9	23.0
BPA	2.7	3.1	3.4	2.3	3.2	2.6	2.8	3.7	2.2*	2.9	2.8
24DCP	1.5	1.7	1.3	1.2	0.9	3.4	0.9*	1.3	1.6	1.1	1.8

a All derived from DEHP.

* *p*-Value < 0.05 for one or more LSMEANS tests between characteristic levels (for race, significance is not indicated if it was found only for Asians).
